# Longitudinal Changes in Gambling, Buying and Materialism in Adolescents: A Population-Based Study

**DOI:** 10.3390/ijerph18062811

**Published:** 2021-03-10

**Authors:** Ana Estévez, Paula Jauregui, Janire Momeñe, Laura Macia, Hibai López-González, Iciar Iruarrizaga, Conchi Riquelme-Ortiz, Roser Granero, Fernando Fernández-Aranda, Cristina Vintró-Alcaraz, Gemma Mestre-Bach, Lucero Munguía, Neus Solé-Morata, Susana Jiménez-Murcia

**Affiliations:** 1 Psychology Department, University of Deusto, 48007 Bilbao, Spain; aestevez@deusto.es (A.E.); paula.jauregui@deusto.es (P.J.); janireml@hotmail.com (J.M.); laura.macia@opendeusto.es (L.M.); hibailopez@ub.edu (H.L.-G.); 2 Departament of Experimental Psychology, Cognitive Processes and Logopedia, Faculty of Social Work, Complutense University of Madrid, 28040 Madrid, Spain; iciariru@psi.ucm.es; 3 Cooperativa Centro Educativo Los Olivos, 30509 Murcia, Spain; presidencia@colegiolosolivos.es; 4 Departament de Psicobiologia i Metodologia, Universitat Autònoma de Barcelona, 08193 Barcelona, Spain; roser.granero@uab.cat; 5 Ciber Fisiopatologia Obesidad y Nutrición (CIBERobn), Instituto Salud Carlos III, 28029 Madrid, Spain; ffernandez@bellvitgehospital.cat (F.F.-A.); cvintro@bellvitgehospital.cat (C.V.-A.); 6 Department of Clinical Sciences, School of Medicine and Health Sciences, University of Barcelona, 08007 Barcelona, Spain; 7 Department of Psychiatry, Bellvitge University Hospital-Institut d’Investigació Biomèdica de Bellvitge (IDIBELL), 08908 Barcelona, Spain; laarcreed_lm@hotmail.com (L.M.); neussm88@gmail.com (N.S.-M.); 8 Facultad de Ciencias de la Salud, Universidad Internacional de la Rioja, 26006 La Rioja, Spain; gemma.mestre@unir.net

**Keywords:** compulsive buying, gambling disorder, materialism, gambling cognitions, adolescence, longitudinal, treatment

## Abstract

Gambling disorder, gambling-related cognitive biases, compulsive buying, and materialistic values lead to impaired functioning in important areas of life. The aims of the present longitudinal study are (1) to evaluate the change produced after one year in those mentioned variables and (2) to examine the gender role in these changes and to analyze the mediational mechanisms among the variables of the study. The sample was composed of 182 adolescents (103 females and 79 males) from secondary education Spanish institutions who completed self-administered questionnaires. Structural equation modeling has been used to explore associations between the different variables. Our results show significant decreases in compulsive buying, materialism, and cognitive biases related to gambling after one year. Gambling disorder severity was directly related to cognitive distortions of gambling and being a man. Compulsive buying was associated with older age and the female gender. Materialism was associated with compulsive buying and the male gender. In conclusion, gambling disorder, gambling-related cognitive biases, compulsive buying, and materialistic values change over time in different ways, according to gender. The understanding of gambling disorder and compulsive buying in adolescents could potentially lead to early prevention and treatment programs for the specific needs of gender and age.

## 1. Introduction

### 1.1. Compulsive Buying

Compulsive buying is characterized by excessive and irrepressible shopping and spending behavior that leads to loss of control and distress or impaired functioning [1,2]. As it shares many characteristics with other addictions [3], some authors refer to it as a behavioral addiction [4,5,6]. It affects approximately 5% of the population, with women showing higher percentages compared to men [7], a prevalence that has been increasing over the years [8]. The average age of onset ranges from 18 to 30 years old [2]. 

Several etiological factors, as well as triggers and maintainers of compulsive buying episodes, have been described. For instance, Trotzke, Starcke, Müller, and Brand reported that exposure to shopping signals generates an unrestrained desire to buy in individuals with compulsive buying that, along with difficulties in making long-term decisions, would explain the maintenance of excessive purchases despite negative consequences [9]. In this sense, people with this behavioral addiction tend to seek rapid and immediate rewards without considering long-term negative consequences [10]. It has also been found that episodes of compulsive buying may be preceded by a state of negative affect [11]. Therefore, this behavior aims to improve mood and low self-esteem [12]. Nicolai and Moshagen claim that an emotional regulation deficit, as well as difficulties in impulse control, may be important risk factors for compulsive buying [13]. Moreover, positive affective states have also been described as triggers for these episodes [14].

### 1.2. Compulsive Buying and Materialism 

Materialistic values have also been highlighted as a predisposing factor to compulsive buying [15]. People with materialistic values consider material possessions or acquisitions as essential to satisfaction and well-being in life. They seek happiness through acquisitions rather than through other means such as personal relationships, experiences, or achievements. Thus, they judge their own and others’ success by the number and quality of possessions. Moreover, they tend to think that through possessions, they will project the desired image of themselves on others [16]. For this reason, materialism has been associated with low subjective well-being, unhappiness, and emotional insecurity [17,18]. In addition, a recent longitudinal study has revealed that materialistic values decrease self-esteem. However, this influence decreases in people with high socioeconomic status and availability of personal resources [19]. Thus, the genesis and maintenance of compulsive buying are related to the role that money and material objects play in families and friends through the symbolic meanings they have adopted [20]. Likewise, the support of parents and peers boosts adolescents’ self-esteem, which, at the same time, decreases their need to appeal to material goods to develop positive and adaptative self-perceptions [21].

### 1.3. Compulsive Buying and Gambling Disorder 

Several studies have focused on the comorbidity between compulsive buying and gambling disorder (GD) [5,22,23,24]. Both disorders share some clinical features, such as loss of control, difficulties in evaluating long-term negative consequences, and deteriorating money management skills, among others [4,5,25,26]. Additionally, materialistic values have also been mentioned as risk factors for the development of gambling disorder in both community and clinical samples [27]. In this sense, a study conducted by Estévez et al. (2020) [28] found that in a clinical sample of young people with gambling disorder, compulsive buying severity was higher for participants who showed higher levels of materialistic values.

Recently, gambling has been established as one of the most addictive behaviors in the adolescent population [29]. Young people engage in gambling behaviors in order to earn money, avoid problems, or increase positive affections, so that adolescence may be a period of special biological and psychological vulnerability [30]. In 2015, in Spain, the prevalence of gambling disorder between 11- and 16-year-old adolescents was estimated at 4.6% [31]. However, this rate has increased in the last few years [32,33]. Some studies uphold that the younger the age of the first gambling experience, the greater the severity of future gambling problems [34]. As opposed to compulsive buying, gambling disorder is more frequent in men than in women [35]. Women start gambling at a later age than men, but they become dependent on gambling more rapidly [36].

### 1.4. Gambling-Related Cognitive Distortions

One of the most noteworthy features of gambling disorder is gambling-related cognitive distortions [37], such as the illusion of control (i.e., the ability to control and predict gains), selective recall of gains, minimization of losses, and irrational thoughts [38,39]. These dysfunctional gambling-related cognitions, along with impulsivity, play an important role in the development, maintenance, and severity of gambling disorder [40,41,42,43]. Previous studies have pointed to the existence of a bidirectional relationship between gambling disorder severity and gambling-related cognitive distortions [44,45]. However, Mallorquí-Bagué et al. did not find associations between cognitive distortions and gambling disorder severity [42]. 

### 1.5. Aims and Hypothesis

Associations between compulsive buying, gambling disorder, materialism, and gambling-related cognitive distortions have been highlighted in some cross-sectional studies. However, to the best of our knowledge, these clinical features have not been studied together in adolescents using a longitudinal design. Specifically, the aims of the present longitudinal study are (1) to evaluate the change produced after one year in gambling disorder severity, gambling-related cognitive distortions, compulsive buying, and materialism in an adolescent sample; (2) to examine the gender role in these changes; (3) to analyze the mediational mechanisms among the different variables of the study. We hypothesize that gambling disorder is expected to be related to the male gender, in contrast to compulsive buying, which is expected to be linked to the female gender. Likewise, older age is expected to be associated with higher gambling disorder severity, compulsive buying, and gambling-related cognitions. 

## 2. Materials and Methods

### 2.1. Participants and Procedure

The community sample analyzed in the study included all participants with complete information in the baseline assessment (Time 1) and in the assessment after one year of follow-up (Time 2; *n* = 182, 103 women (56.6%) and 79 men (43.4%). The mean age of the participants was 16.7 years old (standard deviation (SD) = 2.97), and most of them were born in Spain (164, 90.1%). 

All participants were recruited from secondary education institutions from the Basque Country region in Spain, following convenience sampling. Invitations were sent out to local schools, and a research team member went to the institutions to administer paper-and-pencil questionnaires in their classrooms. The survey included general information concerning the study’s purposes. The adolescents were requested to obtain signed consent from their parents/tutors prior to the study. Participants were reassured of their rights to confidentiality, anonymity, and withdrawal. Furthermore, details of how to contact the research team were handed out.

The evaluation was carried out at two different time points: at the baseline assessment (T1) and the assessment after one year of follow-up (T2). Only those adolescents who completed both assessments (T1 and T2) were included in this study.

### 2.2. Instruments

#### 2.2.1. Gambling Disorder 

The Canadian Adolescent Gambling Inventory (CAGI) [46] was adapted to the Spanish population by Jiménez-Murcia et al. [47]. This is a self-report instrument that measures the adverse psychosocial consequences of gambling and gambling disorder severity behavior in the adolescent population. It is composed of two sections: the first one examines gambling participation in the last three months and includes 20 items that measure the frequency of gambling, with a 6-point response format, and the time dedicated to gambling in a typical week by examining 19 different types of gambling activities. In the second section, CAGI examines the amount of money and articles of value lost due to gambling through 24 items, with a 4-point response format, analyzing five domains: gambling disorder severity problem, psychological consequences, social consequences, financial consequences, and loss of control. It also includes a general problem severity subscale (GPSS), consisting of 9 items distributed across 4 subscales. GPSS is a rating tool for gambling disorder severity and provides a score ranging from 0 to 27. It establishes cut-off points according to the degree of severity of overall gambling: 0–1, no problem with gambling; 2–5, low to moderate severity; 6 or more, high severity. In the present study, Cronbach’s alpha coefficients were 0.91 at Time 1 and 0.94 at Time 2.

The Gambling Related Cognitions Scale (GRCS) [48] was adapted to the Spanish population by Del Prete et al. [37]. The GRCS is an instrument that measures cognition associated with gambling in five different domains: interpretive control/bias, illusion of control, predictive control, gambling-related expectancies, and the perceived inability to stop gambling. It consists of 23 items with a Likert-type scale of 7 points. It shows adequate psychometric indexes, with a Cronbach’s alpha of 0.93 in the total scale and between 0.77 and 0.91 in the different subscales of the original version; in the Spanish adaptation, it shows 0.94 in the total scale and between 0.72 and 0.80 in the different subscales. The internal consistency in the sample of this study for the whole scale was excellent (α = 0.93). Cronbach’s alpha was 0.95 for Time 1 and 0.96 for Time 2.

#### 2.2.2. Compulsive Buying

Pathological Buying Screener (PBS) [49]. The 13-item PBS was translated from English into Spanish by Fernández-Aranda et al. [8] in accordance with the International Test Commission Guidelines for Translating and Adapting Tests [50]. The Spanish version of the PBS was finally reviewed by two other independent Spanish-speaking clinical psychologists who had not been involved in the previous back-translation process. The internal consistency in this study was good (α = 0.86). Cronbach’s alpha for Time 1 was 0.85 and for Time 2 was 0.84. 

### 2.3. Materialism

The Materialism Values Scale (MVS) [16] was adapted to the Spanish population by Lado Couste [51]. This scale is composed of 18 items that assess materialistic values, with an overall score and three subscales measuring importance, success, and happiness based on materialism, following the conceptualization proposed by Richins and Dawson [16]. The items use a four-point Likert scale ranging from 0 (completely disagree) to 3 (completely agree). The Spanish scale has an adequate internal consistency, with a Cronbach’s alpha coefficient of 0.89 for the overall scale and coefficients of 0.77 and 0.83 for the subscales. The Cronbach’s alpha coefficients of the present study ranged between 0.70 and 0.82. Estimated alpha values were 0.79 for the first assessment and 0.81 for the second assessment.

### 2.4. Ethics

The research obtained ethics committee approval from the first author’s university (Ref: ETK-13/15-16) as well as from the Clinical Research Ethics Committee (CEIC) of Bellvitge University Hospital (PSI2011-28349). 

### 2.5. Statistical Analyses

Statistical analysis was carried out with Stata16 for Windows (StataCorp, College Station, TX, USA). The comparison of the raw scores at baseline (T1) and at 1-year of the follow-up (T2), registered in the questionnaires measuring gambling disorder severity (CAGI), gambling-related cognitions (GRCS), compulsive buying (PBS), and materialism (MVS), was performed with *t*-tests for paired samples. Due to the multiple statistical significance tests, the increase in type-I errors was controlled through the Finner method, a familywise error rate stepwise procedure that has proven to be a more powerful test than the classical Bonferroni correction [52].

Path analysis assessed the relationships between the variables of the study. This statistical technique is an extension of multiple regression modeling aimed at estimating the magnitude and significance level of multiple associations within a set of variables, including direct and indirect effects (mediational links) [53]. Path analysis is a useful technique for the analysis of mediation (or mediational) models aimed at identifying and explaining the process that underlies the relationship between an independent variable (X) and a dependent variable (Y) via the effect of a mediator/mediating variable (M). The mathematic scheme is X → M → Y. 

It is used for both exploratory and confirmatory analyses, and, therefore, it allows theory testing and theory development [54]. This work performed path analysis as a case of structural equation modeling (SEM), with maximum-likelihood estimation using the usual fitting indexes (adequate goodness of fit was considered for nonsignificant results in the chi-square test (χ2), root mean square error of approximation (RMSEA) <0.08, Bentler’s comparative fit index (CFI) >0.90, Tucker–Lewis index (TLI) >0.90, and standardized root mean square residual (SRMR) <0.10). The global capacity of the final model was measured with the coefficient of determination (CD).

## 3. Results

### 3.1. Comparison of the Mean Scores at the Two Assessments (T1 and T2)

The number of participants assessed at baseline was *n* = 250 (124 girls versus 126 boys, with a mean age of 18.21 years (SD = 4.88)). Attrition rate (participants’ dropout during the follow-up) was, therefore, 27.2%. To assess the absence of attrition bias, participants with the complete assessment (T1 and T2) and dropouts were compared for the main variables of the study at baseline. No statistical differences were found for sex (χ^2^ = 1.95, *p* = 0.162), education level (χ^2^ = 2.91, *p* = 0.088), GD severity level (CAGI total, T = 0.85, *p* = 0.398), gambling-related cognitive biases (GRCS total, T = 1.49, *p* = 0.137), compulsive buying (PBS total, T = 0.12, *p* = 0.971), and materialism (MVS total, T = 0.039, *p* = 0.844).

**Figure 1:**
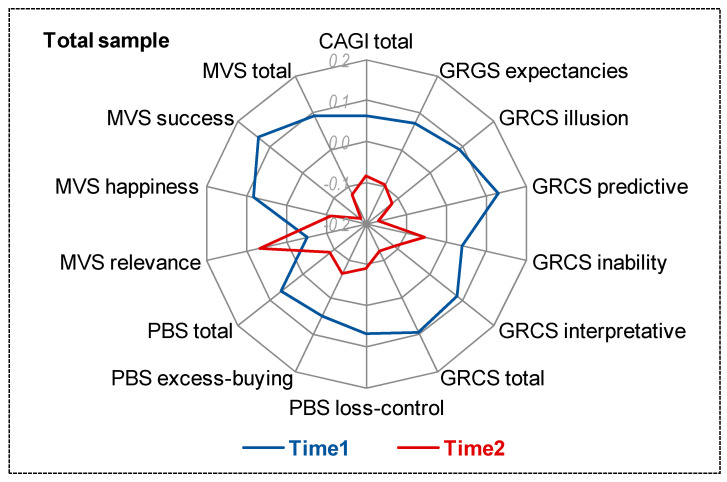
Radar chart (standardized means).CAGI: Canadian Adolescent Gambling Inventory. GRCS: Gambling Related Cognitions Scale. PBS: Pathological Buying Screener. MVS: Materialism Values Scale.

Table 1 shows the changes between the assessment at baseline and at 1-year of the follow-up in the variables of the study for the whole sample (Figure 1 displays the radar chart with the standardized means). Significant decreases were observed in illusion of control, predictive control, global gambling cognitive biases, loss of control, and total scare in the buying measure, the scores in materialism success, and the total score.

**Figure 2:**
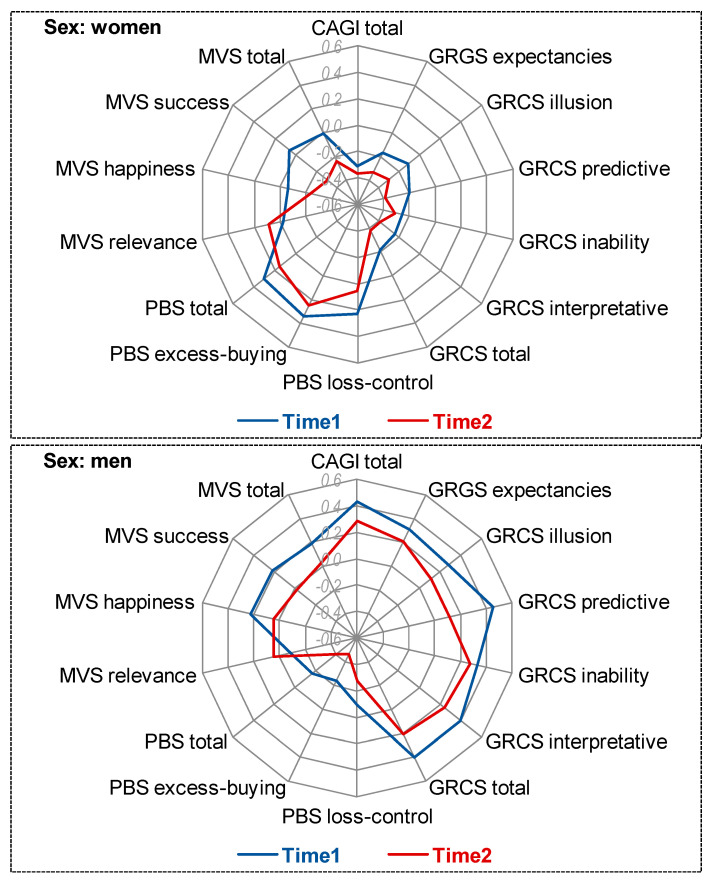
Radar chart stratified by gender (standardized means). CAGI: Canadian Adolescent Gambling Inventory. GRCS: Gambling­­−Related Cognitions Scale. PBS: Pathological Buying Screener. MVS: Materialism Values Scale.

Table 2 shows the assessment of changes stratified by the participants’ gender (Figure 2 displays the radar chart with the standardized means). Among women, significant decreases were obtained in the gambling−related cognitions scale (except for inability to stop gambling) and materialism measures (except in the relevance domain). Among men, significant decreases were only registered for materialism success and total scales.

### 3.2. Path Analysis

**Figure 3:**
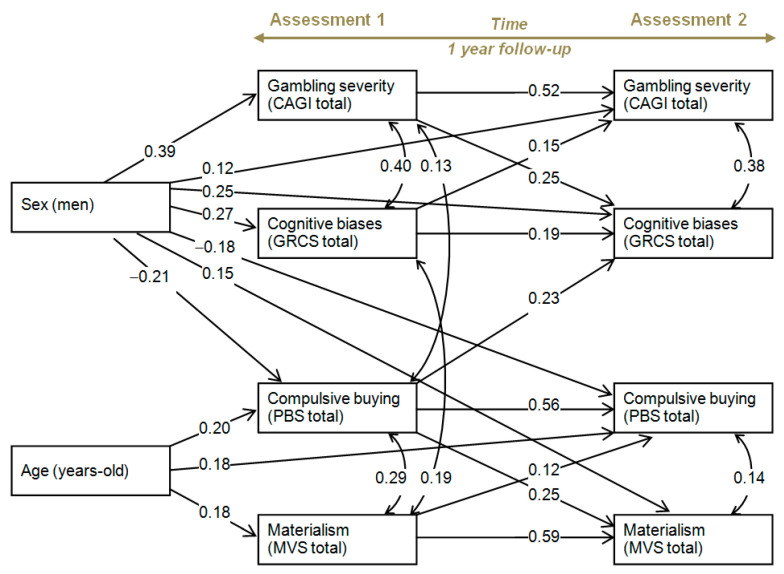
Path diagram with the standardized coefficients. *Note.* Only significant coefficients were retained in the model. Sample size: *n* = 182. GD: gambling disorder. CAGI: Canadian Adolescent Gambling Inventory. GRCS: Gambling-Related Cognitions Scale. PBS: Pathological Buying Screener. MVS: Materialism Values Scale.

An initial model was adjusted with all the direct and indirect effects between the variables of the study. Next, nonsignificant parameters were omitted to obtain a more parsimonious final model for easier interpretation. Table 3 contains the results of the final model, and Figure 3 contains the path diagram with standardized coefficients. Adequate goodness of fit was obtained: χ^2^ = 21.63 (*p* = 0.303), RMSEA = 0.028 (95% confidence interval: 0.001 to 0.073), CFI = 0.995, TLI = 0.989, and SRMR = 0.066. Global predictive capacity was around 41% (CD = 0.411).

At baseline (T1), the male gender was related to gambling disorder severity and gambling-related cognitions, while the female gender was related to compulsive buying. Older age was also related to compulsive buying and materialism at the beginning of the study. At assessment T1, gambling disorder severity was related to gambling-related cognition biases and compulsive buying, and materialism level was related to gambling-related cognitive biases and compulsive buying.

Regarding the follow-up assessment (T2), as expected, each score at T1 in gambling disorder severity, gambling-related cognitions, compulsive buying, and materialism was significant and positively related with the corresponding measure at T2. In addition, subsequent, direct predictive associations were obtained: (a) gambling disorder severity was directly related to the male gender; (b) gambling cognitive distortions were directly related to gambling disorder severity and compulsive buying at baseline; (c) compulsive buying was directly related to the female gender and older age; (d) materialism was directly related to the male gender and compulsive buying at baseline. Some mediational links were also found with gender and age: (a) being a man increased gambling disorder severity at baseline, and the gambling level at the initial assessment was a predictor of gambling disorder severity and cognition biases; (b) being a man also contributed to increases in cognitive biases at the first assessment, and this cognition style contributed to both gambling disorder severity and cognitive biases at Assessment 2; (c) being a woman increased compulsive buying severity at the beginning of the study, which then contributed to compulsive buying and materialism at Assessment 2; (d) older age increased the odds of higher compulsive buying and materialism at baseline, which then contributed to each domain at one year of follow-up. Finally, at the end of the study, gambling disorder severity was related to gambling-related cognitions, and compulsive buying was correlated with materialism.

## 4. Discussion

The first objective of the present study was to evaluate changes in gambling disorder severity, gambling-related cognitive biases, compulsive buying, and materialism after one year of follow-up. The results obtained showed significant decreases in (1) cognitive biases related to gambling (especially those related to the illusion of control and predictive control); (2) compulsive buying (especially loss of control); (3) materialism (especially in the measure of success). 

Regarding gambling behavior, it has been suggested that a period of not gambling for at least one month would be associated with a lower level of cognitive distortions [55]. However, in our sample, the participants were adolescents who may have recently begun to engage in gambling behavior without having developed a moderate or severe gambling problem. In addition, factors such as lack of approval of gambling by people close to them [56], good stress management [57], and good emotional regulation skills [58], among others, may have acted as protective factors in our sample. 

Concerning the decrease in compulsive buying and materialistic values, a possible explanation could be related to the life stage of the participants, characterized by an identity-building process in which personal values play an important role, although they are not yet consolidated and may suffer modifications [59]. In this way, it has been demonstrated that materialistic people tend to make compulsive purchases. However, there are protective factors of compulsive buying in materialistic people that could have influenced the results found [60]. For example, previous studies have highlighted that life satisfaction would act as a protective factor against compulsive buying in students [61]. 

The second aim of this study was to examine the role of gender in the changes described above. Our findings reflect, in the case of females, significant decreases in cognitive biases related to gambling, except for the inability to stop gambling and in materialism, excluding the domain of relevance. For their part, males reported significant decreases in materialism, especially in the measure of success. When compared with males, adolescent females engage in gambling behaviors less frequently, and this may explain the decrease in gambling-related cognitive biases [62]. In addition, it has been proposed that changes in emotional regulation capacity and motivations that occur over the years could influence a decrease in involvement in risk situations [63]. In fact, some studies have pointed out that, during adolescence, it is possible to observe multiple and continuous changes in emotional capacity, including improvements related to affective modulation and discrimination of emotional signals [64]. Likewise, girls usually show higher materialistic values compared to boys [65]. However, these values, as previously mentioned, are built and modified during adolescence [59]. More specifically, it has been observed that materialistic values suffer modifications throughout life in a curvilinear trajectory, in which they are elevated in youth and would decrease towards middle age and increase again towards old age [66]. In this line, previous studies have tested the influence of constructs of communication with friends and the peer effect on the degree of materialism among adolescents [67]. Dependence on the group and the need to get authorization from the group increase materialism, while respect for the group’s decisions decreases it [68].

Finally, the present study aimed to evaluate the mediational mechanisms among the variables analyzed. In the baseline, the male gender was related to gambling disorder severity and gambling-related cognitions, while the female gender was related to compulsive buying. Advancing age was also related to compulsive buying and materialism at the beginning of the study. These results are consistent with previous studies that have noted that compulsive buying is more frequent in women [5,8], while gambling disorder and gambling-related cognitive biases are more frequent in men [56,57]. 

Furthermore, in the present study, gambling disorder severity was related to cognitive biases related to gambling and compulsive buying, and the level of materialism was related to cognitive biases related to gambling and compulsive buying. These results are in line with previous studies, where young adults with gambling disorder have been observed to score higher on cognitive biases related to gambling [69]. In addition, there is comorbidity between gambling disorder severity and compulsive buying [70]. For their part, materialistic values have been considered risk factors for the development of gambling disorder [27] and compulsive buying [59]. 

As for the evaluation of the variables one year later, the scores obtained in the baseline regarding gambling disorder severity, gambling-related cognitions, compulsive buying, and materialism were significant and positively related to the measure corresponding to the year of the follow-up. In addition, direct predictive associations were found: gambling disorder severity was directly related to the male gender, cognitive distortions of gambling with gambling disorder severity, and compulsive buying at baseline. Additionally, compulsive buying was associated with being a woman and being older, and, finally, materialism was associated with being a man and compulsive buying at the baseline. 

At one year of follow-up, gambling disorder severity was associated with cognitive biases related to gambling, and compulsive buying was correlated with materialism. These results are consistent with previous studies that have found a two-way relationship between the severity of gambling disorder and gambling-related cognitive biases [45]. On the other hand, being female and older predicts compulsive buying and materialism, as also found by [71]. Although previous studies have pointed out that materialistic values predict compulsive buying [72], the present study has proven how materialism and compulsive buying show a two-way association. This may be related to the fact that as age advances towards adolescence, the construction of the individual and group “I” is linked to consumption and to what those material objects symbolically represent in terms of prestige, power, and success. In other words, adolescents show a high appreciation for material objects as a way to differentiate themselves from others and be recognized by their peers [73]. In this way, prior research on adolescence and materialism has focused on the importance of the differential effects of age concerning how materialism might be used as a coping strategy for dealing with unpleasant emotions such as loneliness, anxiety, or uncertainly [74,75]. Furthermore, adolescence is understood as a dynamic period of rapid and continuous learning, growth, adaptation, and neurobiological development [76].

### Limitations and Future Research

Despite the strengths of this longitudinal study, there are certain limitations that should be acknowledged. First, this is a sample of adolescents, so social desirability could have skewed the results obtained. Second, the sample has only been collected from one of Spain’s autonomous communities. Future studies could evaluate adolescents from other autonomous communities to ensure greater representativeness. It would also be interesting to replicate the study in other cultural contexts. Third, it should be borne in mind that the results obtained belong to the general population, so they cannot be generalized to the clinical adolescent population. Future studies could analyze these study variables in adolescents with a diagnosis of gambling disorder and/or compulsive buying. Finally, to evaluate each one of the study factors, a self-report questionnaire was used exclusively, so that it is necessary to be extremely cautious when interpreting the results and when talking about gambling disorder and compulsive buying, given that a hetero-applied evaluation by a clinician would also be relevant. However, even if there is a strong need for further investigations in this area, the results of this study could contribute to a better understanding of the variables that may facilitate gambling disorder and compulsive buying in adolescents. Therefore, it could potentially lead to early prevention and treatment programs for specific needs according to gender and age.

## 5. Conclusions

The increase in the incidence and prevalence of behavioral addictions and the relevance of early initiation of the problem of gambling disorder and compulsive buying, together with their serious consequences, make it necessary to better understand these problems in young people and adolescents in order to develop and adapt prevention and treatment programs to specific needs according to gender and age. Furthermore, the understanding of gender-related differences is of great importance in the treatment of behavioral addictions [77]. 

## Figures and Tables

**Table 1:** Changes between Time 1 and Time 2 for the measures of the study.

Variables	Total Sample (*n* = 182)				Pre-Post Changes		
	T1		T2		T-Test Paired Samples		
	*Mean*	*SD*	*Mean*	*SD*	*p*	*95%CI MD*	
*Gambling disorder severity (CAGI)*							
Total score	1.03	2.67	0.75	2.15	0.071	−0.02	0.58
*Gambling biases (GRCS)*							
Gambling expectancies	5.41	3.08	4.97	2.91	0.092	−0.07	0.94
Illusion of control	5.40	2.80	4.84	2.41	0.013 *	0.12	0.99
Predictive control	8.68	5.25	7.46	3.63	0.001 ***	0.47	1.97
Inability to stop gambling	6.34	3.59	6.01	2.99	0.261	−0.25	0.91
Interpretive bias	5.62	3.55	5.17	2.94	0.102	−0.09	0.98
Total score	31.4	16.4	28.5	13.9	0.019 *	0.50	5.44
*Buying (PBS)*							
Loss of control	3.08	3.76	2.55	3.21	0.031 *	0.05	1.01
Excessive buying	3.45	2.71	3.20	2.92	0.161	−0.10	0.59
Total score	6.53	5.89	5.75	5.57	0.034 *	0.06	1.49
*Materialism (MVS)*							
Relevance	9.17	2.78	9.16	3.18	0.981	−0.45	0.46
Happiness	10.03	3.26	9.63	3.42	0.090	−0.06	0.87
Success	11.96	4.51	10.59	4.28	<0.001 ***	0.72	2.00
Total score	31.2	7.78	29.4	8.12	<0.001 ***	0.84	2.70

*Note 1.* SD: standard deviation. GD: gambling disorder. CAGI: Canadian Adolescent Gambling Inventory. GRCS: Gambling−Related Cognitions Scale. PBS: Pathological Buying Screener. MVS: Materialism Values Scale. 95%CI MD: 95% confidence interval for mean difference. * Bold: significant comparison. *Note 2.* Significant comparison means * *p* < 0.05; *** *p* < 0.001.

**Table 2:** Changes between Time 1 and Time 2 for the measures of the study, stratified by gender.

Variables	Girls (*n* = 103)				Pre-Post Changes			Boys (*n* = 79)				Pre-Post Changes		
	T1		T2		T-Test Paired			T1		T2		T-Test Paired		
	*Mean*	*SD*	*Mean*	*SD*	*p*	*95%CI MD*		*Mean*	*SD*	*Mean*	*SD*	*p*	*95%CI MD*	
*Gambling disorder severity (CAGI)*														
Total score	0.13	0.48	0.04	0.19	0.083	−0.01	0.19	2.20	3.71	1.67	3.03	0.131	−0.16	1.23
*Gambling biases (GRCS)*														
Gambling expectancies	4.80	2.09	4.21	0.88	0.004 **	0.20	0.97	6.20	3.89	5.96	4.11	0.654	−0.82	1.30
Illusion of control	4.91	2.40	4.35	1.45	0.024 *	0.07	1.05	6.03	3.16	5.48	3.16	0.172	−0.24	1.33
Predictive control	7.46	3.68	6.37	1.40	0.002 **	0.41	1.77	10.27	6.47	8.87	4.93	0.067	−0.10	2.89
Inability stop gambling	5.46	1.87	5.15	0.69	0.122	−0.08	0.71	7.48	4.80	7.13	4.23	0.572	−0.89	1.60
Interpretive bias	4.70	2.28	4.25	1.15	0.037 *	0.03	0.87	6.81	4.46	6.37	3.97	0.433	−0.68	1.56
Total score	27.3	11.0	24.3	4.65	0.005 **	0.90	5.04	36.8	20.4	33.8	19.2	0.250	−2.13	8.05
*Compulsive Buying (PBS)*														
Loss of control	3.59	3.70	3.03	3.37	0.063	−0.03	1.16	2.41	3.74	1.92	2.88	0.236	−0.32	1.28
Excessive buying	4.26	2.54	4.17	3.02	0.724	−0.40	0.58	2.39	2.56	1.94	2.23	0.067	−0.03	0.94
Total score	7.85	5.73	7.20	5.87	0.174	−0.29	1.59	4.80	5.66	3.86	4.54	0.100	−0.18	2.06
*Materialism (MVS)*														
Relevance	9.08	2.72	9.24	3.17	0.578	−0.75	0.42	9.29	2.87	9.06	3.22	0.538	−0.51	0.96
Happiness	9.77	3.23	9.09	3.51	0.023 *	0.10	1.26	10.38	3.28	10.34	3.19	0.921	−0.72	0.80
Success	11.43	4.13	9.93	3.91	0.001 ***	0.63	2.36	12.65	4.91	11.46	4.60	0.017 *	0.22	2.16
Total score	30.3	7.70	28.3	8.47	0.002 **	0.74	3.27	32.3	7.78	30.9	7.43	0.040 *	0.07	2.84

*Note 1.* SD: standard deviation. GD: gambling disorder. CAGI: Canadian Adolescent Gambling Inventory. GRCS: Gambling­­−Related Cognitions Scale. PBS: Pathological Buying Screener. MVS: Materialism Values Scale. 95%CI MD: 95% confidence interval for mean difference. * Bold: significant comparison. *Note 2.* Significant comparison means * *p* < 0.05; ** *p* < 0.01; *** *p* < 0.001.

**Table 3:** Path analysis: direct, indirect, and total effects.

Direct Effects	Variables	Coeff	SE	z	*p*	StdCoeff
GD severity—T1	Gender (male)	2.0763	0.3649	5.69	<0.001 ***	0.3886
GD severity—T2	GD severity—T1	0.4153	0.0516	8.04	<0.001 ***	0.5208
	Cognitive biases—T1	0.0192	0.0083	2.33	0.020 *	0.1477
	Materialism—T1	0.0324	0.0144	2.25	0.024 *	0.1183
	Gender (male)	0.5210	0.2541	2.05	0.040 *	0.1223
Cognitive biases—T2	GD severity—T1	1.2724	0.3883	3.28	0.001 ***	0.2460
	Cognitive biases—T1	0.1606	0.0601	2.67	0.008 **	0.1902
	CB—T1	0.5418	0.1434	3.78	<0.001 ***	0.2333
	Gender (male)	6.9858	1.9728	3.54	<0.001 ***	0.2528
Cognitive biases—T1	Gender (male)	8.8473	2.3049	3.84	<0.001 ***	0.2705
Materialism—T1	Age (years-old)	0.4757	0.1859	2.56	0.010 **	0.1827
Materialism—T2	Materialism—T1	0.6091	0.0574	10.61	<0.001 ***	0.5909
	CB—T1	0.3307	0.0781	4.24	<0.001 ***	0.2451
	Gender (male)	2.3640	0.8934	2.65	0.008 **	0.1472
CB—T1	Gender (male)	−3.7296	0.8037	−4.64	<0.001 ***	−0.3133
	Age (years-old)	0.4046	0.1385	2.92	0.003 **	0.2034
CB—T2	CB—T1	0.5310	0.0550	9.65	<0.001 ***	0.5613
	Gender (male)	−1.9862	0.6477	−3.07	0.002 **	−0.1764
	Age (years-old)	0.3317	0.1053	3.15	0.002 **	0.1763
Indirect effects		Coeff	SE	z	*p*	StdCoeff
GD severity—T2	Gender (male)	1.0326	0.2018	5.12	<0.001 ***	0.2423
	Age (years-old)	0.0154	0.0091	1.69	0.091	0.0216
Cognitive biases—T2	Gender (male)	2.0427	1.3183	1.55	0.121	0.0739
	Age (years-old)	0.2192	0.0949	2.31	0.021 *	0.0475
Materialism—T2	Gender (male)	−1.2333	0.3942	−3.13	0.002 **	−0.0768
	Age (years-old)	0.4236	0.1387	3.05	0.002 **	0.1578
CB—T2	Gender (male)	−1.9806	0.4736	−4.18	<0.001 ***	−0.1759
	Age (years-old)	0.2148	0.0769	2.8	0.005 **	0.1142
Total effects		Coeff	SE	z	*p*	StdCoeff
GD severity—Time1	Gender (male)	2.0763	0.3649	5.69	<0.001 ***	0.3886
GD severity—Time2	GD severity—Time 1	0.4153	0.0516	8.04	<0.001 ***	0.5208
	Cognitive biases—T1	0.0192	0.0083	2.33	0.020 *	0.1477
	Materialism—T1	0.0324	0.0144	2.25	0.024 *	0.1183
	Gender (male)	1.5536	0.2916	5.33	<0.001 ***	0.3646
	Age (years-old)	0.0154	0.0091	1.69	0.091	0.0216
Cognitive biases—T2	GD severity—T1	1.2724	0.3883	3.28	0.001 ***	0.2460
	Cognitive biases—T1	0.1606	0.0601	2.67	0.008 **	0.1902
	CB—T1	0.5418	0.1434	3.78	<0.001 ***	0.2333
	Gender (male)	9.0285	1.9231	4.69	<0.001 ***	0.3267
	Age (years-old)	0.2192	0.0949	2.31	0.021 *	0.0475
Cognitive biases—T1	Gender (male)	8.8473	2.3049	3.84	<0.001 ***	0.2705
Materialism—T1	Age (years-old)	0.4757	0.1859	2.56	0.010 **	0.1827
Materialism—T2	Materialism—T1	0.6091	0.0574	10.61	<0.001 ***	0.5909
	CB—T1	0.3307	0.0781	4.24	<0.001 ***	0.2451
	Gender (male)	1.1307	0.8898	1.27	0.204	0.0704
	Age (years-old)	0.4236	0.1387	3.05	0.002 **	0.1578
CB—T1	Gender (male)	−3.7296	0.8037	−4.64	<0.001 ***	−0.3133
	Age (years-old)	0.4046	0.1385	2.92	0.003 **	0.2034
CB—T2	CB—T1	0.5310	0.0550	9.65	<0.001 ***	0.5613
	Gender (male)	−3.9668	0.7532	−5.27	<0.001 ***	−0.3523
	Age (years-old)	0.5465	0.1264	4.32	<0.001 ***	0.2904

*Note 1.* SE: standard error. GD: gambling disorders. CB: compulsive buying. StdCoeff: standardized coefficients. Sample size: *n* = 182. *Note 2.* Significant comparison means * *p* < 0.05; ** *p* < 0.01; *** *p* < 0.001.

## Data Availability

The data presented in this study are available on request from the corresponding author. The data are not publicly available due to ethical restrictions in order to protect the confidentiality of the participants.
